# Effect of body mass index and disc degeneration on lumbar biomechanics after unilateral biportal endoscopy–unilateral laminectomy bilateral decompression for lumbar spinal stenosis: a finite element study

**DOI:** 10.3389/fbioe.2026.1752088

**Published:** 2026-06-30

**Authors:** Tusheng Li, Jingbo Ma, Aobo Wang, Ziqian Ma, Yu Ding, Lei Zang

**Affiliations:** 1 Department of Orthopedics, Beijing Chaoyang Hospital, Capital Medical University, Beijing, China; 2 Orthopedics of TCM Senior Department, The Sixth Medical Center of People’s Liberation Army General Hospital, Beijing, China

**Keywords:** body mass index, disc degeneration, finite element study, lumbar biomechanics, lumbar spinal stenosis, unilateral biportal endoscopy–unilateral laminectomy bilateral decompression

## Abstract

**Objective:**

To evaluate the effects of body mass index (BMI) and disc degeneration on lumbar biomechanics following unilateral biportal endoscopy–unilateral laminectomy bilateral decompression (UBE-ULBD) for lumbar spinal stenosis (LSS).

**Methods:**

Computed tomography data from a 32-year-old healthy male volunteer (height: 1.75 m, weight: 70 kg, BMI: 22.86 kg/m^2^) were used to construct an intact L3–S1 finite-element (FE) model in ANSYS APDL version 13.0. Based on this model, a UBE-ULBD surgical model was created at the L4–L5 segment. Four BMI levels (22.86, 26.12, 29.39, and 32.65 kg/m^2^) and three disc states (normal, mild degeneration, and severe degeneration) were simulated. Biomechanical outcomes included segmental range of motion (ROM), intradiscal pressure (IDP), and facet joint stress (FJS). Model validity was verified by comparing ROM and degeneration-related changes with previously published *in vitro* and FE findings.

**Results:**

Compared with the intact model, the UBE-ULBD model showed a stable and consistent pattern of biomechanical variation across motion directions under different BMI levels and disc degeneration grades. Single-factor analyses showed that within the same degeneration grade, increasing BMI generally increased ROM, IDP, and FJS at the L4–L5 segment. At a fixed BMI, progressive disc degeneration was associated with decreased ROM and IDP but stepwise increases in FJS, indicating posterior column overload. Mixed-effects analyses revealed that in degenerated discs, a higher BMI partially offset the degeneration-related decreases in ROM and IDP; however, ROM remained lower than that of the intact model, whereas IDP values only shifted from decreased to slightly increased in severe degeneration cases. FJS exhibited a cumulative and amplifying response to BMI and disc degeneration, especially during extension and rotation. The combination of severe disc degeneration and high BMI produced peak posterior element loading under multidirectional loading, suggesting that weight gain with disc degeneration globally amplifies spinal loading and markedly aggravates posterior column overload.

**Conclusion:**

FE analysis showed that disc degeneration primarily alters load distribution (i.e., reduced ROM and IDP but increased FJS), whereas BMI increases the overall load magnitude (i.e., simultaneous increases in ROM, IDP, and FJS at any given grade of degeneration). Their interaction produces a peak posterior column overload pattern under the severe degeneration + high BMI condition. Clinically, body weight and disc degeneration status should be simultaneously considered. During UBE-ULBD, posterior stabilizing structures should be preserved as much as possible, and weight management along with postoperative core muscle rehabilitation should be emphasized.

## Introduction

Lumbar spinal stenosis (LSS) is a common cause of low back and leg pain in the elderly, the prevalence of which increases with age (Lurie and Tomkins-Lane, 2016; Zaina et al., 2016). An estimated 103 million people suffer from LSS worldwide, with reports showing its prevalence to be as high as 11% among American adults (Katz et al., 2022). Surgery is typically recommended for patients with LSS who are refractory to conservative treatment. Conventional open decompression is a classic procedure for LSS, but the relatively extensive iatrogenic trauma it causes has long been a concern for spine surgeons (Scholler et al., 2017; Chiu et al., 2020). In recent years, with the development of minimally invasive concepts and innovations in surgical technology, minimally invasive spine surgery has increasingly been recognized as an alternative to open lumbar procedures for LSS while offering several advantages such as reduced soft-tissue damage, faster recovery, and shorter hospital stays (Pan et al., 2020).

In 2016, Choi et al. (2016) first utilized unilateral biportal endoscopy (UBE) for the treatment of LSS and confirmed its safety and effectiveness. Since then, UBE has been widely used as an effective minimally invasive technique for LSS (Kaen et al., 2023; Wang et al., 2023). Unlike uniportal endoscopy, UBE separates the working and observation channels, both of which are independent and can be freely adjusted during surgery. This separation allows for better flexibility and a wider field of view, thereby facilitating comprehensive decompression of the spinal canal (Jiang et al., 2022; Zheng et al., 2022). Clinically, when stenosis is confined to one side, unilateral decompression using UBE usually yields satisfactory outcomes. However, for patients with bilateral stenosis and/or severe central canal stenosis, bilateral decompression is often required, commonly performed as UBE–unilateral laminectomy bilateral decompression (UBE-ULBD) (Hua et al., 2022). Several studies have shown favorable clinical results with UBE-ULBD for the treatment of LSS (Hua et al., 2022; Zhang et al., 2025). Although UBE-ULBD is performed through a minimally invasive approach, adequate bilateral decompression usually still requires targeted resection of posterior elements, including parts of the lamina, ligamentum flavum, and, when necessary, the medial facet. Therefore, despite its tissue-sparing advantages over conventional open surgery, this procedure may still alter postoperative segmental load transfer and stability, particularly in patients with pre-existing degenerative changes.

Endoscopic decompression better preserves lumbar stability than does open surgery, mainly because it enables precise decompression with reduced bone and soft-tissue resection (Sriphirom et al., 2021; Hwang et al., 2024). Nevertheless, multiple factors can still influence postoperative clinical outcomes. Numerous studies have indicated that advanced disc degeneration and elevated body mass index (BMI) are risk factors for poorer outcomes and postoperative complications in patients with degenerative lumbar disease (Kong et al., 2020; Fan et al., 2021; Jitpakdee et al., 2023). Progressive disc degeneration can cause uneven load distribution, loss of disc height, reduced shock-absorbing capacity of the spine, and accelerated facet joint degeneration (Inoue and Espinoza, 2011; Iorio et al., 2016). Over time, these changes can severely disrupt the “three-column” mechanical balance and ultimately cause spinal canal stenosis and recurrent symptoms (Inoue and Espinoza, 2011; Iorio et al., 2016). Elevated BMI increases spinal loading and alters the dynamics of discs, vertebrae, surrounding ligaments, and muscles (Samartzis et al., 2012; Jentzsch et al., 2015; Ghezelbash et al., 2017; Coppock et al., 2021). Increased lumbar load can aggravate disc degeneration, cause abnormal stress distribution in the vertebral body leading to fractures, and increase facet joint loading, thereby accelerating facet degeneration (Samartzis et al., 2012; Jentzsch et al., 2015; Ghezelbash et al., 2017; Coppock et al., 2021). Furthermore, obese patients often have weak lumbar stabilizing muscles and increased fatty infiltration, which predisposes them to the risk of lumbar instability (Samartzis et al., 2012; Ozcan-Eksi et al., 2021). Therefore, understanding how varying degrees of disc degeneration and BMI affect lumbar biomechanics is imperative.

Finite-element (FE) analysis is a reliable method for evaluating spinal biomechanics given its ability to digitally simulate spinal material properties, morphological structures, boundary conditions, and loading conditions and enable repeated simulations of mechanical tests that cannot be directly performed *in vivo* (Cai et al., 2020; Zhang et al., 2024). Consequently, FE analysis has been considered a preclinical research tool with important implications for optimizing patient selection and improving surgical strategies. Previous FE studies on disc mechanical failure have often emphasized tissue-level mechanisms, particularly annulus fibrosus stress–strain behavior (Zhou et al., 2021; Zhou et al., 2023). However, segment-level biomechanical responses after surgical interventions, especially after UBE-ULBD under different BMI and disc degeneration conditions, remain insufficiently investigated. Furthermore, most previous studies have focused on BMI or disc degeneration as a single factor and thus fail to fully capture the combined effects of “structural factors” (disc degeneration) and “load factors” (axial loading) (Cai et al., 2020; Wang et al., 2024; Zhang et al., 2024).

In the current study, we constructed a parameterized L3–S1 FE model and simulated UBE-ULBD at the L4–L5 segment. We then systematically analyzed lumbar biomechanics under different disc degeneration grades and BMI levels and evaluated their mixed effects. This study aimed to provide biomechanical evidence to inform preoperative risk stratification, refine surgical decision-making, and guide postoperative rehabilitation strategies for patients undergoing UBE-ULBD.

## Materials and methods

### Study design and FE model construction

This study used lumbar computed tomography (CT) images from a 32-year-old healthy male volunteer (height, 1.75 m; weight, 70 kg; BMI, 22.86 kg/m^2^) as the basis for model construction. Informed consent was obtained and the study was approved by the Ethics Committee of the Committee of the Beijing Chaoyang Hospital, Capital Medical University (No. 2025-KE-962). A three-dimensional FE model of the L3–S1 spine was established to simulate lumbar biomechanics after UBE-ULBD under various BMI levels and disc degeneration grades. The model was constructed using ANSYS APDL version 13.0 (ANSYS, United States), following the methodology described by Viceconti et al. (Viceconti et al., 2005).

**FIGURE 1:**
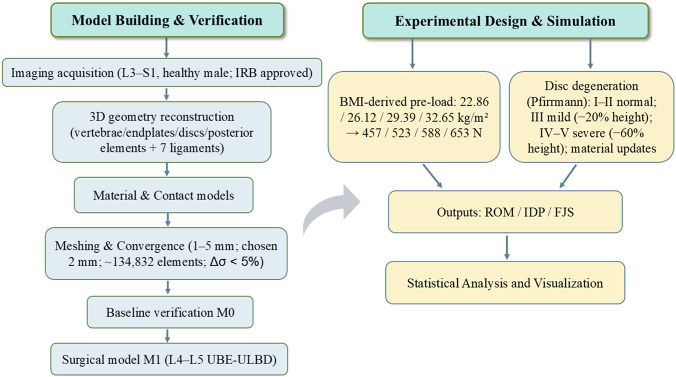
Flowchart of research design.

The geometric model included vertebral bodies (cortical bone, cancellous bone, and facet joint cartilage), posterior bony elements (spinous processes, pedicles, transverse processes, and facet joints), intervertebral discs (annulus fibrosus, nucleus pulposus, and endplates), and seven major ligaments, namely, the anterior longitudinal ligament, posterior longitudinal ligament, ligamentum flavum, supraspinal ligament, interspinous ligament, intertransverse ligament, and facet capsular ligament. A schematic of the overall modeling workflow is presented in Figure 1. The material properties of the various components were assigned according to published data (Polikeit et al., 2003; Bresnahan et al., 2009; Wu et al., 2023; Wu et al., 2024; Ma et al., 2025; Ma et al., 2025) and are summarized in Table 1.

**TABLE 1:** Material properties in the present FE model.

Structure	Young’s modulus (MPa)	Poisson’s ratio	Cross-sectional area (mm^2^)	Average length
Vertebrae				
Cortical bone	12,000.0	0.2	-	-
Cancellous bone	340.0	0.2	-	-
Facet joint cartilage	10.0	0.4	-	-
Disc				
Endplates	24.0	0.4	-	-
Nucleus pulposus	1.0	0.5	-	-
Annulus fibrosus	500.0	0.5	-	-
Ligaments				
ALL	7.8 (<12.0%) 20.0 (>12.0%)	0.4	63.7	20.0
PLL	10.0 (<11.0%) 20.0 (>11.0%)	0.3	20.0	12.0
SSL	8.0 (<20.0%) 15.0 (>20.0%)	0.3	70.0	22.0
ISL	10.0 (<14.0%) 11.6 (>14.0%)	0.3	70.0	13.0
LF	15.8 (<6.2%) 19.5 (>6.2%)	0.3	40.0	15.0
ITL	10.0 (<18.0%) 58.4 (>18.0%)	0.3	1.8	32.0
FCL	7.5 (<25.0%) 32.9 (>25.0%)	0.3	30.0	5.0

ALL, anterior longitudinal ligament; PLL, posterior longitudinal ligament; SSL, supraspinal ligament; ISL, interspinous ligament; LF, ligamentum flavum; ITL, intertransverse ligament; FCL, facet capsular ligament.

The disc was modeled using a composite structure, with the annulus fibrosus and nucleus pulposus occupying 56% (674 mm^2^) and 44% (539 mm^2^) of the cross-sectional area, respectively. The annulus fibrosus was modeled using an anisotropic material to reflect its lamellar structure, whereas the nucleus pulposus was assumed to be nearly incompressible. Degeneration effects were modeled by modifying disc geometry and material properties, without incorporating fluid–solid coupling or swelling behavior. Facet joint surfaces were modeled using low-friction sliding contacts with a friction coefficient of 0.1, allowing limited relative motion between articular surfaces, whereas cartilaginous interfaces were modeled using bonded contacts. All ligaments were modeled using nonlinear tension–only elements. Second-order reduced-integration elements were used for the ligaments. Remote points were created on the superior surfaces of the L3–S1 vertebral bodies to facilitate load application and determination of biomechanical responses. A full-factorial design was adopted to systematically evaluate all combinations of BMI levels and degeneration grades, enabling direct assessment of their combined biomechanical effects.

### Mesh generation and convergence testing

Mesh generation was performed using second-order tetrahedral elements in the ANSYS Workbench. Several element sizes ranging from 1 to 5 mm were tested. Under a 10 N⋅m flexion moment, the von Mises stress in the L4–L5 segment nucleus pulposus was calculated to evaluate mesh sensitivity. When the element size was decreased from 2 mm to 1 mm, the stress difference was only 1.45%, which met the accuracy requirements. Therefore, an element size of 2 mm was used in subsequent simulations. The final intact model (M0) contained 134,832 elements.

To further verify mesh independence, convergence analysis was conducted under 500 N axial compression combined with 6 N⋅m flexion and 6 N⋅m extension moments (Wilke et al., 1994). The convergence criterion was a <5% change in the target stress between two successive mesh refinements, which was calculated using the following formula:

$$\Delta \%=\frac{\left|\sigma_{n}-{\sigma_{n}}^{-1}\right|}{{\sigma_{n}}^{-1}}\times 100\;\%$$

**FIGURE 2:**
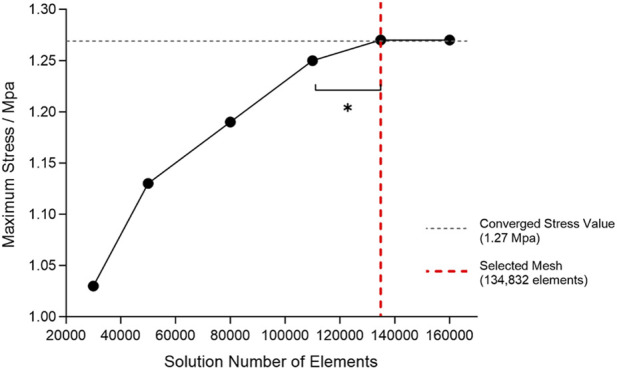
Mesh convergence validation for maximum stress in the FE model.

The test results indicated that when the total element number reached approximately 134,800, the change in stress was <5%, and the stress values stabilized, confirming mesh convergence. Therefore, this mesh generation scheme was adopted for subsequent FE analysis (Figure 2).

### UBE-ULBD surgical model

**FIGURE 3:**
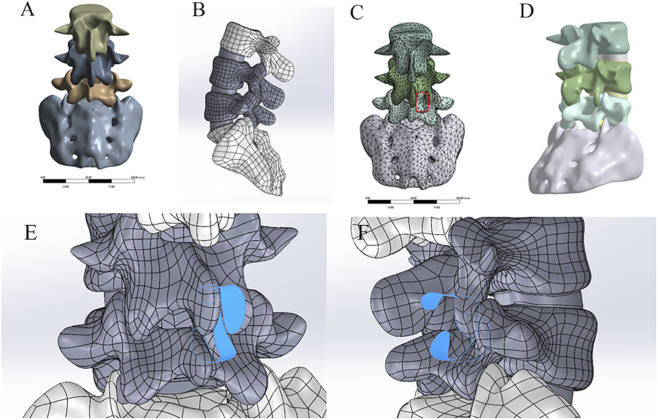
Schematic diagrams of the M0 and M1 models, along with the surgical approach boundaries for the M1 model. **(A,B)** Presentation of the M0 model; **(C,D)** Presentation of the M1 model (The red box indicates the laminotomy approach); **(E,F)** Presentation of the surgical approach boundaries (In Figure E, the highlighted blue area represents the resection plane of the facet joints; in Figure F, the highlighted blue area represents the resection plane at the base of the spinous process).

UBE-ULBD was simulated at the L4–L5 segment, with the right-sided approach being modeled. After identifying the surgical segment, two independent channels were created: an observation channel for the endoscope and a working channel for instruments. Under endoscopic visualization, ipsilateral laminotomy decompression was first performed, followed by resection of the hyperplastic facet joints and hypertrophic ligamentum flavum to decompress the ipsilateral nerve root and dural sac. Thereafter, the endoscope was tilted toward the contralateral side, and part of the spinous process base and of the inner edge of the contralateral lamina were removed to allow contralateral canal decompression. Depending on the degree of stenosis, the contralateral ligamentum flavum was partially resected to decompress the contralateral nerve root and dural sac. In the present model, only the medial portion of the ipsilateral facet joint was partially resected, whereas the contralateral facet joint was preserved. To balance decompression adequacy and segmental stability, the extent of ipsilateral facetectomy was limited to a stability-preserving range, with more than 50% of the ipsilateral facet joint retained. The resulting postoperative model was defined as the UBE-ULBD model (M1). Schematic diagrams of the M0 and M1 models, along with the surgical approach boundaries for the M1 model, are shown in Figure 3.

### Loading and boundary conditions

Loading and boundary conditions were based on established experimental studies (Yamamoto et al., 1989; Chen et al., 2001). All degrees of freedom of the S1 vertebra were fixed as boundary constraints. An axial compressive load was applied on the superior endplate of L3 and was combined with ±10 N⋅m pure moments to simulate six typical physiological motions: flexion, extension, left lateral bending, right lateral bending, left rotation, and right rotation. All loads were uniformly distributed across the loading surface *via* remote control points to ensure uniformity and consistency of moment and displacement throughout the simulation process.

### BMI-related loading conditions

Using the volunteer’s height (1.75 m) as a reference, four body weights were simulated: 70, 80, 90, and 100 kg, corresponding to BMI values of 22.86, 26.12, 29.39, and 32.65 kg/m^2^, respectively. The S1 vertebra was fixed to restrict its displacement. Two-thirds of body weight was applied as a vertical compressive load on the superior endplate of L3, corresponding to 457, 523, 588, and 653 N for the four body weights, respectively (Zhang et al., 2024).

### Disc degeneration models

Disc degeneration was classified according to the Pfirrmann and Thompson grading systems. Accordingly, grades I–II were considered normal discs, grade III represented mild degeneration, and grades IV–V were categorized as severe degeneration (Zhang and Han, 2023). Based on previous literature (Ruberte et al., 2009; Zhang and Han, 2023), disc degeneration was simulated by reducing disc height and nucleus pulposus area. Specifically, disc height was reduced by 20% and 60% to simulate for mild and severe degeneration, respectively. To maintain total disc volume, the annulus fibrosus material was used to replace the reduced nucleus pulposus volume. The material parameters of the endplates and discs were also adjusted to reflect endplate sclerosis and changes in tissue properties during degeneration (Table 2).

**TABLE 2:** Material properties assigned to different tissues in degenerated disc models.

Structure	Young’s modulus (MPa)	Poisson’s ratio
Mild degeneration		
Cartilage endplate	24	0.4
Osteophytes	100	0.2
Soft tissue	Hyper-elastic material, C1 = 0.4, C2 = 0.1	
Annulus ground	Hyper-elastic material, C1 = 0.4, C2 = 0.1	
Nucleus pulposus	Hyper-elastic material, C1 = 0.14, C2 = 0.035	
Severe degeneration		
Cartilage endplate	100	0.4
Osteophytes	100	0.2
Soft tissue	Hyper-elastic material, C1 = 0.9, C2 = 0.23	
Annulus ground	Hyper-elastic material, C1 = 0.9, C2 = 0.23	
Nucleus pulposus	Hyper-elastic material, C1 = 0.19, C2 = 0.045	

By modifying the elastic modulus, water content, and stiffness of the nucleus pulposus and annulus fibrosus according to the Pfirrmann grade, FE models representing mild and severe disc degeneration were constructed. To validate the disc degeneration models, the range of motion (ROM) at the L4–L5 segment in the degeneration models under a 10 N⋅m pure moment was compared with experimental data reported by Mimura et al. (Mimura et al., 1994).

### Observation indicators

Biomechanical data for the L4–L5 segment in the M0 model under normal conditions (i.e., BMI of 22.86 kg/m^2^ and normal intervertebral discs) were collected. Using this baseline as a reference, we evaluated how the M1 model responded at the L4–L5 segment under different BMI levels (22.86, 26.12, 29.39, and 32.65 kg/m^2^) and disc states (normal, mild degeneration, and severe degeneration). The following outcomes were analyzed to assess the effects of BMI and disc degeneration on lumbar biomechanics after UBE-ULBD: segmental ROM, intradiscal pressure (IDP), and facet joint stress (FJS). To assess the combined effects of disc degeneration and BMI, we compared biomechanical outcomes across all 12 modeled combinations (4 BMI levels × 3 degeneration grades) in the postoperative UBE-ULBD model.

## Results

### Validation of the intact model

**FIGURE 4:**
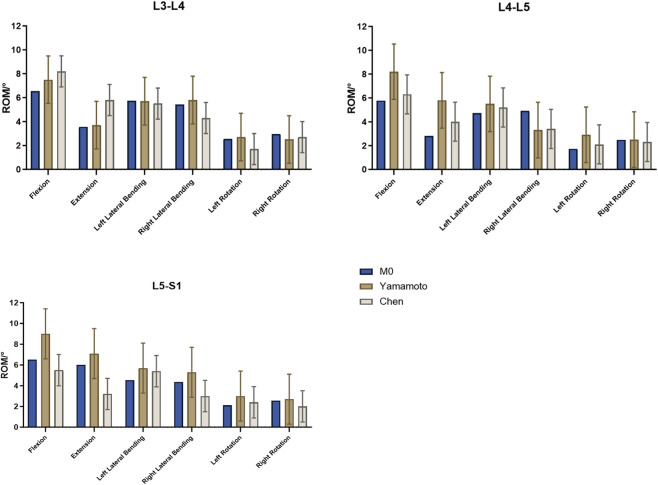
Validation of the M0 model and comparison with previous literature.

To ensure reliable predictive performance, the M0 model was first validated. Under a 150 N axial load combined with ±10 N⋅m pure moments, ROM values at each segment were extracted and compared with the experimental data reported by Yamamoto and Chen et al. (Yamamoto et al., 1989; Chen et al., 2001). Notably, we found that the ROM values of the current M0 model closely matched those reported in the literature, with errors within 8% in all motion directions, indicating that the model had good kinematic accuracy and applicability (Figure 4).

### Validation of the disc degeneration models

**FIGURE 5:**
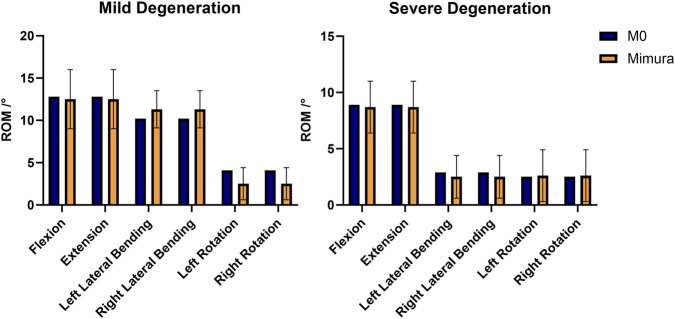
Validation of the disc degeneration model and comparison with previous literature.

In mild and severe degenerative states, ROM values in the models fell within the standard deviation of the experimental results reported by Mimura et al. (1994) (Figure 5). These findings indicate that the lumbar degeneration models created in the current study were properly calibrated and can serve as a reliable basis for subsequent biomechanical analyses of the M1 model under different BMI and degeneration conditions.

### UBE-ULBD model under different BMI and disc degeneration conditions

#### Single-factor effects

Within the same degeneration grade, an increase in BMI generally promoted an increasing trend in ROM, IDP, and FJS at the L4–L5 segment, suggesting that higher body weight amplifies segmental motion and loading on the anterior and posterior columns. Among the three indices, FJS was the most sensitive to BMI. When BMI was 32.65 kg/m^2^ with normal discs, FJS increased by approximately 37.8%–50.0% across different motion directions compared to the M0 model. With mild and severe disc degeneration, FJS increased by 45.9%–61.8% and by 55.1%–75.0%, respectively.

At any given BMI, progressive disc degeneration was associated with a decrease in ROM and IDP but a stepwise increase in FJS at the L4–L5 segment. In severely degenerated discs, FJS increased by 23.5%–46.1%, 33.7%–55.7%, 43.9%–65.9%, and 55.1%–75.0% at a BMI of 22.86, 26.12, 29.39, and 32.65 kg/m^2^, respectively, across various motion directions compared to the M0 model.

### Mixed effects of BMI and disc degeneration

In degenerated discs, an increase in BMI partially mitigated the degeneration-related decrease in ROM and caused a shift in IDP values from decreased to slightly increased in the severe disc degeneration state, suggesting that external loading can partially “pull back” the motion and pressure response of degenerated discs. However, overall ROM still remained lower than that in the M0 model.

In contrast, BMI and degeneration severity promoted a cumulative increase in FJS. In other words, the more severe the degeneration and the higher the BMI, the greater the posterior FJS, particularly during extension and rotation. In fact, the combination of severe degeneration and high BMI produced the highest posterior column overload pattern across all directions. This mixed effect indicates that on a degenerative background, increased BMI amplifies the overall mechanical response and markedly intensifies posterior load concentration, providing a biomechanical basis for persistent low back pain, accelerated facet joint degeneration, and increased adjacent-segment burden after surgery.

**FIGURE 6:**
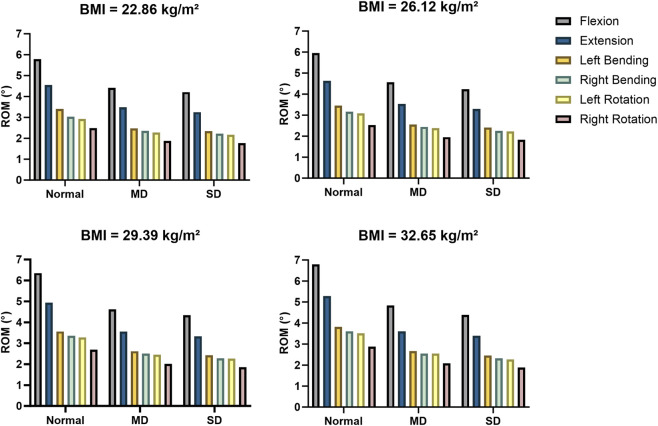
Results of the ROM values for different M1 models.

**FIGURE 7:**
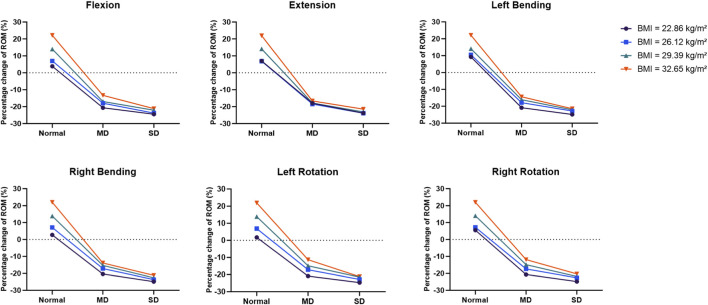
Results of ROM growth rates for different M1 models compared to the M0 model.

**FIGURE 8:**
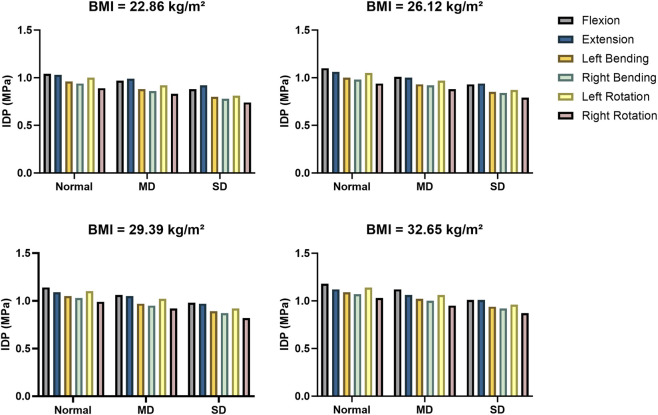
Results of the IDP values for different M1 models.

**FIGURE 9:**
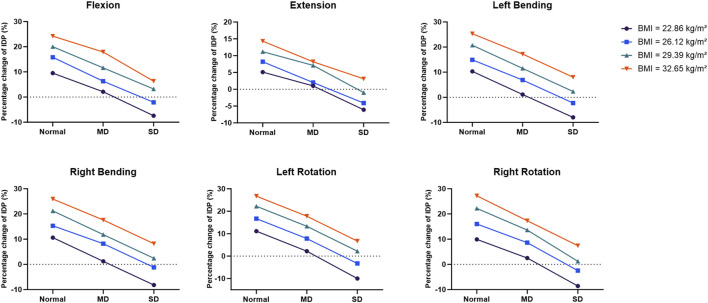
Results of IDP growth rates for different M1 models compared to the M0 model.

**FIGURE 10:**
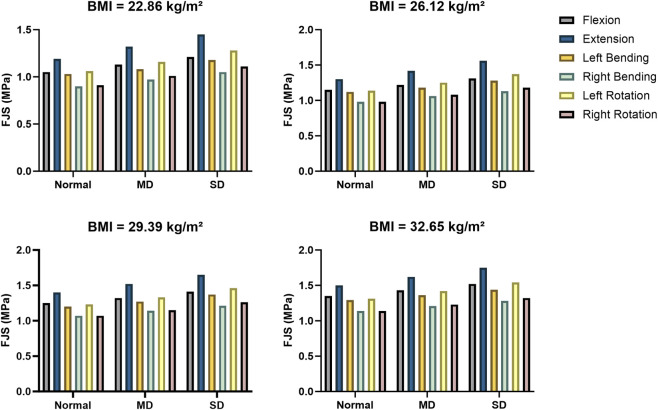
Results of the FJS values for different M1 models.

**FIGURE 11:**
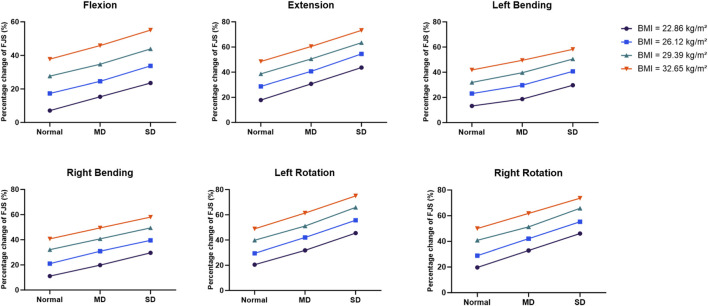
Results of FJS growth rates for different M1 models compared to the M0 model.

Detailed data on ROM, IDP, and FJS for the M1 model under different BMI and disc degeneration conditions, as well as their percentage changes relative to the M0 model, are summarized in Tables 3–5 and illustrated in Figures 6–11.

**TABLE 3:** Results of ROM values for different M1 models and their growth rates compared to the M0 model.

Models	Grading	BMI (kg/m^2^)	FL (°)	EX (°)	LB (°)	RB (°)	LR (°)	RR (°)
M0	Normal	22.86	5.57	4.33	3.12	2.95	2.88	2.36
M1	Normal	22.86	5.79 (+3.9%)	4.55 (+5.1%)	3.41 (+9.3%)	3.03 (+2.7%)	2.93 (+1.7%)	2.49 (+5.5%)
	MD	22.86	4.42 (−20.6%)	3.48 (−19.6%)	2.47 (−20.8%)	2.35 (−20.3%)	2.28 (−20.8%)	1.87 (−20.8%)
	SD	22.86	4.21 (−24.4%)	3.25 (−24.9%)	2.34 (−25.0%)	2.22 (−24.7%)	2.17 (−24.7%)	1.77 (−25.0%)
	Normal	26.12	5.96 (+7.0%)	4.63 (+6.9%)	3.45 (+10.6%)	3.16 (+7.1%)	3.08 (+6.9%)	2.53 (+7.2%)
	MD	26.12	4.57 (−18.0%)	3.53 (−18.5%)	2.56 (−17.9%)	2.44 (−17.3%)	2.38 (−17.4%)	1.95 (−17.4%)
	SD	26.12	4.24 (−23.9%)	3.30 (−23.8%)	2.41 (−22.8%)	2.25 (−23.7%)	2.22 (−22.9%)	1.83 (−22.5%)
	Normal	29.39	6.35 (+14.0%)	4.94 (+14.1%)	3.56 (+14.1%)	3.36 (+13.9%)	3.28 (+13.9%)	2.69 (+14.0%)
	MD	29.39	4.62 (−17.1%)	3.55 (−18.0%)	2.62 (−16.0%)	2.50 (−15.3%)	2.45 (−14.9%)	2.01 (−14.8%)
	SD	29.39	4.34 (−22.1%)	3.33 (−23.1%)	2.43 (−22.1%)	2.28 (−22.7%)	2.26 (−21.5%)	1.85 (−21.6%)
	Normal	32.65	6.80 (+22.1%)	5.28 (+21.9%)	3.81 (+22.1%)	3.60 (+22.0%)	3.51 (+21.9%)	2.88 (+22.0%)
	MD	32.65	4.83 (−13.3%)	3.61 (−16.6%)	2.67 (−14.4%)	2.54 (−13.9%)	2.55 (−11.5%)	2.08 (−11.9%)
	SD	32.65	4.39 (−21.2%)	3.40 (−21.5%)	2.45 (−21.5%)	2.32 (−21.4%)	2.27 (−21.2%)	1.88 (−20.3%)

MD: mild degeneration; SD: severe degeneration; FL: flexion; EX: extension; LB: left bending; RB: right bending; LR: left rotation; RR: right rotation; The growth rate (%) represents the comparison between the M1 model and the M0 model under different conditions.

**TABLE 4:** Results of IDP values for different M1 models and their growth rates compared to the M0 model.

Models	Grading	BMI (kg/m^2^)	FL (MPa)	EX (MPa)	LB (MPa)	RB (MPa)	LR (MPa)	RR (MPa)
M0	Normal	22.86	0.95	0.98	0.87	0.85	0.90	0.81
M1	Normal	22.86	1.04 (+9.5%)	1.03 (+5.1%)	0.96 (+10.3%)	0.94 (+10.6%)	1.00 (+11.1%)	0.89 (+9.9%)
	MD	22.86	0.97 (+2.1%)	0.99 (+1.0%)	0.88 (+1.1%)	0.86 (+1.2%)	0.92 (+2.2%)	0.83 (+2.5%)
	SD	22.86	0.88 (−7.4%)	0.92 (−6.1%)	0.80 (−8.0%)	0.78 (−8.2%)	0.81 (−10.0%)	0.74 (−8.6%)
	Normal	26.12	1.10 (+15.8%)	1.06 (+8.2%)	1.00 (+14.9%)	0.98 (+15.3%)	1.05 (+16.7%)	0.94 (+16.0%)
	MD	26.12	1.01 (+6.3%)	1.00 (+2.0%)	0.93 (+6.9%)	0.92 (+8.2%)	0.97 (+7.8%)	0.88 (+8.6%)
	SD	26.12	0.93 (−2.1%)	0.94 (−4.1%)	0.85 (−2.3%)	0.84 (−1.2%)	0.87 (−3.3%)	0.79 (−2.5%)
	Normal	29.39	1.14 (+20.0%)	1.09 (+11.2%)	1.05 (+20.7%)	1.03 (+21.2%)	1.10 (+22.2%)	0.99 (+22.2%)
	MD	29.39	1.06 (+11.6%)	1.05 (+7.1%)	0.97 (+11.5%)	0.95 (+11.8%)	1.02 (+13.3%)	0.92 (+13.6%)
	SD	29.39	0.98 (+3.2%)	0.97 (−1.0%)	0.89 (+2.3%)	0.87 (+2.4%)	0.92 (+2.2%)	0.82 (+1.2%)
	Normal	32.65	1.18 (+24.2%)	1.12 (+14.3%)	1.09 (+25.3%)	1.07 (+25.9%)	1.14 (+26.7%)	1.03 (+27.2%)
	MD	32.65	1.12 (+17.9%)	1.06 (+8.2%)	1.02 (+17.2%)	1.00 (+17.6%)	1.06 (+17.8%)	0.95 (+17.3%)
	SD	32.65	1.01 (+6.3%)	1.01 (+3.1%)	0.94 (+8.0%)	0.92 (+8.2%)	0.96 (+6.7%)	0.87 (+7.4%)

MD: mild degeneration; SD: severe degeneration; FL: flexion; EX: extension; LB: left bending; RB: right bending; LR: left rotation; RR: right rotation; The growth rate (%) represents the comparison between the M1 model and the M0 model under different conditions.

**TABLE 5:** Results of FJS values for different M1 models and their growth rates compared to the M0 model.

Models	Grading	BMI (kg/m^2^)	FL (MPa)	EX (MPa)	LB (MPa)	RB (MPa)	LR (MPa)	RR (MPa)
M0	Normal	22.86	0.98	1.01	0.91	0.81	0.88	0.76
M1	Normal	22.86	1.05 (+7.1%)	1.19 (+17.8%)	1.03 (+13.2%)	0.90 (+11.1%)	1.06 (+20.5%)	0.91 (+19.7%)
	MD	22.86	1.13 (+15.3%)	1.32 (+30.7%)	1.08 (+18.7%)	0.97 (+19.8%)	1.16 (+31.8%)	1.01 (+32.9%)
	SD	22.86	1.21 (+23.5%)	1.45 (+43.6%)	1.18 (+29.7%)	1.05 (+29.6%)	1.28 (+45.5%)	1.11 (+46.1%)
	Normal	26.12	1.15 (+17.3%)	1.30 (+28.7%)	1.12 (+23.1%)	0.98 (+21.0%)	1.14 (+29.5%)	0.98 (+28.9%)
	MD	26.12	1.22 (+24.5%)	1.42 (+40.6%)	1.18 (+29.7%)	1.06 (+30.9%)	1.25 (+42.0%)	1.08 (+42.1%)
	SD	26.12	1.31 (+33.7%)	1.56 (+54.5%)	1.28 (+40.7%)	1.13 (+39.5%)	1.37 (+55.7%)	1.18 (+55.3%)
	Normal	29.39	1.25 (+27.6%)	1.40 (+38.6%)	1.20 (+31.9%)	1.07 (+32.1%)	1.23 (+39.8%)	1.07 (+40.8%)
	MD	29.39	1.32 (+34.7%)	1.52 (+50.5%)	1.27 (+39.6%)	1.14 (+40.7%)	1.33 (+51.1%)	1.15 (+51.3%)
	SD	29.39	1.41 (+43.9%)	1.65 (+63.4%)	1.37 (+50.5%)	1.21 (+49.4%)	1.46 (+65.9%)	1.26 (+65.8%)
	Normal	32.65	1.35 (+37.8%)	1.50 (+48.5%)	1.29 (+41.8%)	1.14 (+40.7%)	1.31 (+48.9%)	1.14 (+50.0%)
	MD	32.65	1.43 (+45.9%)	1.62 (+60.4%)	1.36 (+49.5%)	1.21 (+49.4%)	1.42 (+61.4%)	1.23 (+61.8%)
	SD	32.65	1.52 (+55.1%)	1.75 (+73.3%)	1.44 (+58.2%)	1.28 (+58.0%)	1.54 (+75.0%)	1.32 (+73.7%)

MD: mild degeneration; SD: severe degeneration; FL: flexion; EX: extension; LB: left bending; RB: right bending; LR: left rotation; RR: right rotation; The growth rate (%) represents the comparison between the M1 model and the M0 model under different conditions.

## Discussion

LSS is a common orthopedic condition characterized by herniated disc, hyperplastic facet joints, and hypertrophic ligamentum flavum, which induce spinal canal narrowing, nerve root and/or cauda equina compression, and various clinical symptoms such as intermittent claudication and low back/leg pain (Lurie and Tomkins-Lane, 2016; Zaina et al., 2016; Katz et al., 2022). Surgery is recommended for patients who are refractory to conservative management. With advances in instruments and endoscopic technology, minimally invasive endoscopic spine surgery has become an effective option for treating LSS (Pan et al., 2020). For LSS patients with bilateral stenosis and/or severe central canal stenosis, UBE-ULBD has been increasingly used to achieve adequate decompression, with evidence showing satisfactory clinical outcomes (Hua et al., 2022; Zhang et al., 2025).

Compared to unilateral decompression, ULBD requires more extensive resection of the bone and ligaments, including the base of the spinous process, inner edge of the contralateral lamina, contralateral ligamentum flavum, and contralateral facet joint (Spetzger et al., 1997; Hua et al., 2022; Zhang et al., 2025), which may have a greater impact on lumbar stability (Hartmann et al., 2012; Ding et al., 2024). In an FE study, Ding et al. (2024) demonstrated that the interlaminar approach combined with ULBD for LSS preserves relatively stable biomechanics, although their analysis was conducted under the assumption of normal disc and body weight conditions. However, these conditions do not fully reflect real-world scenarios wherein patients often present with disc degeneration and varying BMI levels. Numerous clinical studies have linked advanced disc degeneration and high BMI to poor postoperative improvement and increased incidence rates of complications (Fan et al., 2021; Jitpakdee et al., 2023; Kong et al., 2020). Consequently, the influence of BMI and disc degeneration on lumbar biomechanics has received increasing attention. However, biomechanical studies on UBE-ULBD under different disc degeneration and/or BMI conditions remain limited.

The results of our FE analysis revealed that the biomechanical contributions of disc degeneration and BMI were not identical. At a fixed BMI, progressive disc degeneration promoted a decrease in ROM and IDP but induced a marked increase in FJS, indicating that degenerated discs operate in a mechanical state of increased stiffness, relative unloading of the anterior column, and chronic overload of the posterior column. In contrast, within the same degeneration grade, increasing BMI promoted an in increase in ROM, IDP, and FJS, demonstrating that higher body weight amplifies segmental motion and loading in the anterior and posterior columns. Their interaction revealed that posterior overload peaks under the severe degeneration + high BMI condition. FJS exhibited a synergistic amplification effect with both factors such that the more severe the disc degeneration and the higher the BMI, the greater the FJS, especially during extension and rotation.

Taken together, disc degeneration determines how stiff the segment is and how loads are distributed between the anterior and posterior columns, whereas BMI determines how much overall load is applied to the already degenerated structure. Under severe disc degeneration and high BMI conditions, the lumbar segment may enter a complex mechanical environment characterized by no obvious increase, or even a slight restriction, in ROM but with highly concentrated local stress and chronic overload of posterior structures. Therefore, our results provide theoretical support for incorporating weight management into the comprehensive prevention and treatment of degenerative lumbar disease and highlight the importance of preserving or reconstructing posterior stabilizing structures during UBE-ULBD while focusing on core stability and extensor endurance training and avoiding early large-amplitude extension/rotation movements that produce high post-surgical posterior stress.

Disc degeneration shifts load from the anterior to the posterior column, making the facet joints the key load-bearing points. On this basis, elevated BMI further increases the overall load magnitude, inducing a synergistic amplification effect on FJS, particularly during extension and rotation. Facet joints are crucial for maintaining segmental stability, and sharp increases in FJS indicate a tendency toward facet-mediated instability (Inoue and Espinoza, 2011). Clinically, the articular cartilage and capsule of the posterior facet joints are rich in nociceptive nerve endings (Kalichman and Hunter, 2007). Chronic high stress can trigger cartilage wear, synovitis, and osteophyte formation, which correspond radiographically to facet joint hypertrophy, sclerosis, and degeneration and clinically to mechanical low back pain aggravated by prolonged sitting, extension, and rotation (Kalichman and Hunter, 2007; Perolat et al., 2018). The results of our FE analysis thus provide a reasonable biomechanical explanation for facet joint-origin low back pain in the context of degeneration.

It should also be noted that the present study was intended to evaluate segment-level postoperative biomechanics after UBE-ULBD rather than disc tissue-level failure mechanisms. Although annulus fibrosus stress–strain distributions are important for understanding disc mechanical failure and herniation risk (Zhou et al., 2021; Zhou et al., 2023), ROM, IDP, and FJS were selected as the primary outcome measures in the current study because they more directly reflect postoperative motion and load transfer at the operated segment. Accordingly, annulus fibrosus stress–strain distributions were not analyzed in the present FE framework.

Our findings emphasize that under the same external load, degenerated segments exhibited reduced ROM but increased FJS. This phenomenon does not represent a genuine increase in stability but rather a form of pseudo-stability that depends on posterior constraint: nucleus dehydration, annular fibrosis, and endplate sclerosis all increase the effective stiffness of the disc, reduce the compliance of the anterior column, and limit overall displacement (Adams and Roughley, 2006; Galbusera et al., 2014). To complete the necessary fine coupled motions, the system passively recruits posterior structures (articular cartilage, joint capsule, and facet joints) to provide more constraint and guidance, shifting the contact area posteriorly and increasing the combined compressive and shear load per unit area (Rohlmann et al., 2006; Inoue and Espinoza, 2011; Cai et al., 2020). Clinically, this pseudo-stability can manifest as mechanical low back pain induced by extension and rotation, accompanied by facet joint degeneration/sclerosis and ligamentum flavum hypertrophy (Kalichman and Hunter, 2007; Perolat et al., 2018; Cai et al., 2020). Previous studies have shown that facet joint–mediated pain is often aggravated during extension and rotation, whereas ligamentum flavum hypertrophy is closely associated with chronic mechanical stress (Fukuyama et al., 1995; Kosaka et al., 2007).

Therefore, in decompression-dominated procedures, such as UBE-ULBD, a balance between sufficient decompression and preservation of posterior load-bearing capacity must be maintained. Excessive resection of the medial facet joint and capsular structures should be avoided to minimize adverse effects on facet joint loading and segmental stability. Prior studies have shown that progressive facet joint resection sequentially weakens segmental stability and that overly aggressive resection can promote clear instability (Abumi et al., 1990; Lee et al., 2004). Moreover, biomechanical studies have suggested that in the presence of degeneration, low bone mass, and high BMI, segments may present with a pseudo-stability phenotype in which ROM does not markedly increase but internal stress increases sharply, consistent with the pattern observed in this study (Ma et al., 2025).

In the current study, we constructed a parameterized lumbar FE model based on a single subject and systematically examined the combined effects of structural factors (disc degeneration) and load factors (axial loading approximated according to the BMI). This approach minimizes inter-subject geometric variability, allows for a more direct assessment of the relative contributions of each factor, and provides a modeling and analytical framework for future multi-case, patient-specific FE studies. However, given that the model geometry is based on a single individual, the conclusions primarily reflect trends and relative comparisons under that geometry and may not be directly generalizable to the broader population without external validation. Furthermore, as this study did not involve repeated observations from multiple independent samples, linear mixed-effects modelling was not performed. Consequently, the combined effect of BMI and disc degeneration was explained through a comparison of biomechanical factors across all simulated conditions.

This study has several limitations. First, the FE model did not incorporate active muscle forces, and loading was applied using pure moments under simplified boundary conditions. As a result, passive spinal structures, including the intervertebral disc, ligaments, and facet joints, may bear a greater proportion of the applied load than they would *in vivo*, which may influence the absolute magnitude of segmental motion and internal stress responses. This limitation may be particularly relevant in obese individuals, in whom paraspinal muscle degeneration and fatty infiltration may further alter physiological load-sharing. Second, although the simulated decompression was performed with preservation of more than 50% of the ipsilateral facet joint and without direct resection of the contralateral facet joint, the exact resected volume or percentage of each posterior structure was not separately quantified, which may limit the computational reproducibility of the surgical model. Third, degeneration-related disc swelling behavior was not considered, and fluid–solid interactions that may affect intradiscal pressure were simplified. Fourth, BMI was modeled as an increase in axial compressive load only, consistent with prior finite element studies using BMI-dependent loading conditions (Zhang et al., 2024); however, obesity-related postural changes, including anterior shift of the center of mass and the associated additional flexion moment, were not incorporated. Therefore, the present model is better suited for evaluating the effect of increased axial loading magnitude than for fully reproducing the sagittal loading mechanics of obesity. Fifth, the model geometry was based on a young, healthy subject and therefore did not incorporate age-related anatomical features commonly seen in LSS patients, such as altered lumbar lordosis, facet hypertrophy, and reduced bone quality. This may limit the direct generalizability of the facet joint stress findings to elderly clinical populations. Sixth, this study focused purely on biomechanical analysis without direct clinical correlation or validation. Additionally, although obesity-level BMI conditions were included, more severe obesity levels were not modeled. Moreover, because the model geometry was derived from a single male volunteer, sex-specific anatomical and biomechanical differences were not considered, which may limit the generalizability of the findings. Future multicenter clinical and basic research is needed to more accurately characterize the biomechanical and clinical impacts of disc degeneration and BMI on outcomes after UBE-ULBD for LSS.

## Conclusion

FE analysis demonstrated that progressive disc degeneration promotes a posterior column overload pattern at the involved segment, which is characterized by decreased ROM and IDP but increased FJS. BMI elevation, at any given degeneration grade, generally amplifies ROM, IDP, and FJS. Through their interaction, the severe degeneration + high BMI conduction produces peak posterior column loading. In other words, disc degeneration primarily determines load distribution, whereas BMI determines the overall load magnitude. These findings suggest that during UBE-ULBD, posterior stabilizing structures should be preserved as much as possible and that perioperative weight management and postoperative core muscle rehabilitation should be prioritized, especially in patients with severe degeneration and elevated BMI. Although this study provides FE-based evidence for understanding lumbar loading patterns in obese patients with degenerative disease, additional experimental data are needed to further validate these results.

## Data Availability

The raw data supporting the conclusions of this article will be made available by the authors, without undue reservation.
